# Construction of Drug Network Based on Side Effects and Its Application for Drug Repositioning

**DOI:** 10.1371/journal.pone.0087864

**Published:** 2014-02-04

**Authors:** Hao Ye, Qi Liu, Jia Wei

**Affiliations:** 1 R&D Information, AstraZeneca, Shanghai, China; 2 School of Life Sciences and Technology, Tongji University, Shanghai, China; Koç University, Turkey

## Abstract

Drugs with similar side-effect profiles may share similar therapeutic properties through related mechanisms of action. In this study, a drug-drug network was constructed based on the similarities between their clinical side effects. The indications of a drug may be inferred by the enriched FDA-approved functions of its neighbouring drugs in the network. We systematically screened new indications for 1234 drugs with more than 2 network neighbours, 36.87% of the drugs achieved a performance score of **N**ormalized **D**iscounted **C**umulative **G**ain in the top **5** positions (NDCG@5)≥0.7, which means most of the known FDA-approved indications were well predicted at the top 5 positions. In particular, drugs for diabetes, obesity, laxatives and antimycobacterials had extremely high performance with more than 80% of them achieving NDCG@5≥0.7. Additionally, by manually checking the predicted 1858 drug-indication pairs with **E**xpression **A**nalysis **S**ystematic **E**xplorer (EASE) score≤10^−5^ (EASE score is a rigorously modified Fisher exact test p value), we found that 80.73% of such pairs could be verified by preclinical/clinical studies or scientific literature. Furthermore, our method could be extended to predict drugs not covered in the network. We took 98 external drugs not covered in the network as the test sample set. Based on our similarity criteria using side effects, we identified 41 drugs with significant similarities to other drugs in the network. Among them, 36.59% of the drugs achieved NDCG@5≥0.7. In all of the 106 drug-indication pairs with an EASE score≤0.05, 50.94% of them are supported by FDA approval or preclinical/clinical studies. In summary, our method which is based on the indications enriched by network neighbors may provide new clues for drug repositioning using side effects.

## Introduction

The inefficiency of pharmaceutical drug development with high expenditure but low productivity has been widely discussed [1], [2], [3], [4]. Drug repositioning is considered to be a promising strategy to revitalize the slowing drug discovery pipeline due to shorter development timelines and lower risk of unexpected toxicity [5], [6], [7]. Traditionally, most of the successful examples mainly relied on serendipity or ‘happy accidents’ (eg, Viagra, Dapoxetine, Duloxetine) [6], [8], which made repositioning very unpredictable. In 2006, Lamb et al [9] proposed the connectivity map based on the gene expression profiles of drugs for repositioning, which is the first computational method in this field. Then a group of investigators utilized structural features of compounds/proteins to predict new targets of drugs, such as molecular docking [10], [11], QSAR modelling [12]. In addition, the association between diseases/drugs in genetic activity was suggested to facilitate repositioning, such as genome-wide association [13], pathway profiles [14], [15], and transcriptional responses [16]. Furthermore, several integrative methods which combined chemical or genetic features were proposed to predict the drug targets or indications, for example, PREDICT [17], TMFS [11]. Obviously, most of these methods focus on the molecular mechanism of action (MOA) from a genotypic perspective. Nevertheless, the pre-clinical outcomes based on MOA often do not correlate well with therapeutic efficacy in drug development. It is estimated that of all the compounds effective in cell assays, only 30% of them could work in animals. Even worse, only 5% of them could work in humans [18].

The gap between MOA and the physiological responses of drugs may limit the usefulness of the methods mentioned above. Side effects are generated when the drugs bind to off-targets, which perturb unexpected metabolic or signaling pathways [19]. Therefore, side effects from clinical patients may be seen as valuable read-outs of drug effects on human bodies, which may also serve as a promising perspective for drug repositioning. Up to now, only a few of the repositioning efforts focus on physiological responses. Most of them are developed using the side effect data in SIDER [20], which was constructed by the Bork' group in 2010. The latest version of SIDER contains 996 drugs and 4192 side effects. Lun proposed *DRoSEf* [21],where the basic hypothesis is that if the side effects associated with a drug are also induced by many drugs treating a disease, then this drug should be evaluated as a candidate to treat that disease. Initially, they constructed side effect profiles of diseases based on drug-indication data and drug-side effect data. The QSAR models were trained to build associations between structures and side effects. Then indications of the drug could be predicted by combining the side effect profiles of diseases with structure and side effect associations. Lun's work pioneered an approach to drug repositioning using side effects and achieved good performance. Up to now, side effects are still the best data available to reflect disease characteristics even if they are not equivalent to pathological symptoms of diseases (for example, side effects may be generated by off-targets, which are not the accompanying disorders of the disease). In addition, the limited quantities of drugs in SIDER may impact the QSAR training processes in *DRoSEF*, which has only worked well in 145 diseases. With expanded coverage of the drugs, *DRoSEF* could achieve more promising performance.

In this study, we intend to propose a network based method for drug repositioning by exploring the entire existing catalog of side effect data. Instead of directly building the relationship between side effects and diseases, we would like to construct drug-drug relationships through side effect similarities. Our basic hypothesis is that drugs with similar side-effect profiles may also share similar therapeutic properties [22]. A drug network could be constructed based on the similarities of side effects. In this way, the indications of a drug may be predicted by the functional distribution of its neighbouring drugs. Since we have already investigated chemical structures [23] and pathway profiles [14], [15] for drug repositioning, side effect based repositioning could enhance our computational repositioning platform and provide complementary evidence.

## Materials and Methods

### Drug side effects

In this study, side effects were extracted from *Meyler*'*s Side Effects of Drugs 15^th^ edition* [24]. Additional drug sides effects, especially for the drugs launched after 2006, were collected from *Side Effects of Drugs Annuals* (2007–2012) [25] and the FDA drug approval package (see **Table 1**). Specifically, each electronic book was converted from PDF to text format by Acrobat professional v10.1. Then, a Java program was implemented to parse the drug information and side effects. Considering the side effects in *Meyler*'*s Side Effects of Drugs 15^th^ edition* and *Side Effects of Drugs Annuals* (2007–2012) were organised using MedDRA vocabularies version 15.1, the *preferred items* (PT level) in MedDRA were utilized as the standard side effect vocabulary. The side effect data from other resources were mapped to MedDRA *preferred items*, avoiding the semantic redundancy. For example “*respiratory diseases*” and “*respiration diseases*” were identified as two different side effects in the raw data, and both of them were mapped to the same *preferred item* “*respiratory disease*” in MedDRA.

**Table 1:** Drug side effects resources.

Source	Description
Meyler's Side Effects of Drugs (15^th^ edition)	The International Encyclopaedia of Adverse Drug Reactions, a publication for a history spanning more than 60 years. It has been published every four years since 1980. It is the most comprehensive and authoritative resource of drug side effects, which contains>3,400 drugs and>12,600 side effects.
*Side Effects of Drugs Annuals(2007*–*2012)*	The Side Effects of Drugs Annual was first published in 1977. It has been continually published since then as a yearly update to the encyclopaedia *Meyler*'*s Side Effects of Drugs*.
Drugs@FDA Database	Drugs@FDA includes most of the drug products approved by the FDA since 1939. Most patient information, labels, approval letters, reviews, approval packages and other information for drug products approved since 1998 are available.
SIDER	Contains information on 996 marketed drugs and corresponding 4192 recorded adverse drug reactions. The information is extracted from public documents and package inserts.

### External test samples

As described in **Table 1**, SIDER is a frequently cited resource for drug side effects. Herein, 98 drugs in SIDER version 2 which were not covered by the drug network were used as external test samples (see the 98 drugs in **Table S1**). Also, the side effects in SIDER were mapped to *preferred items* in MedDRA version 15.1.

### Drug indication

FDA-approved indications were obtained from *Citeline Pipeline*, *Thomson Reuters Partnering* and *GeneGo* (see details in **Table 2**). Next, each indication was modified to a MeSH heading. Finally, we obtained 2183 drugs with 6495 clinical side effects and 994 4^th^ level MeSH items.

**Table 2:** Drug indication resources.

Source	Description
Pipeline	Developed by Citeline. Reputed to have collected information on drugs developed for any disease anywhere in the world since 1980, including their approval dates, companies and related clinical trials.
Thomson Reuters Partnering	The database was formally called IDdb and acquired by Thomson Reuters. The drug pipeline information is integrated from a variety of sources, such as company websites, and over 200 global conferences.
GeneGO	GeneGO is a comprehensive biological database, covering a wide range of data, including pathways, drug information, biomarkers etc. Each drug indication is mapped to a MeSH item.

### Drug network construction with side effects (see Figure 1)

#### Step 1: Build the side effect fingerprint

Herein, each side effect was treated as a feature vector. If a drug displays side effect *i*, then it would be tagged “1” in the element *i*, otherwise, it would be marked “0”. After that a 0-1 binary vector was defined as the side effect fingerprint for the drug. For the 2183 drugs with recorded clinical side effect data, each would be assigned a 6495-dimension vector.

#### Step 2: Calculate the similarity between drugs

The Jaccard index was used to evaluate the similarities of side effect fingerprints. In a given drug pair (labelled A and B), the Jaccard index for the binary vectors could be calculated as the formula I.
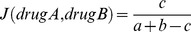
(I)


In this formula, a, b represent the number of side effects for drugs A and B, respectively. c represents the number of side effects shared by drug A and B.

#### Step 3: Evaluate the side effect similarities between drugs

**Figure 1:**
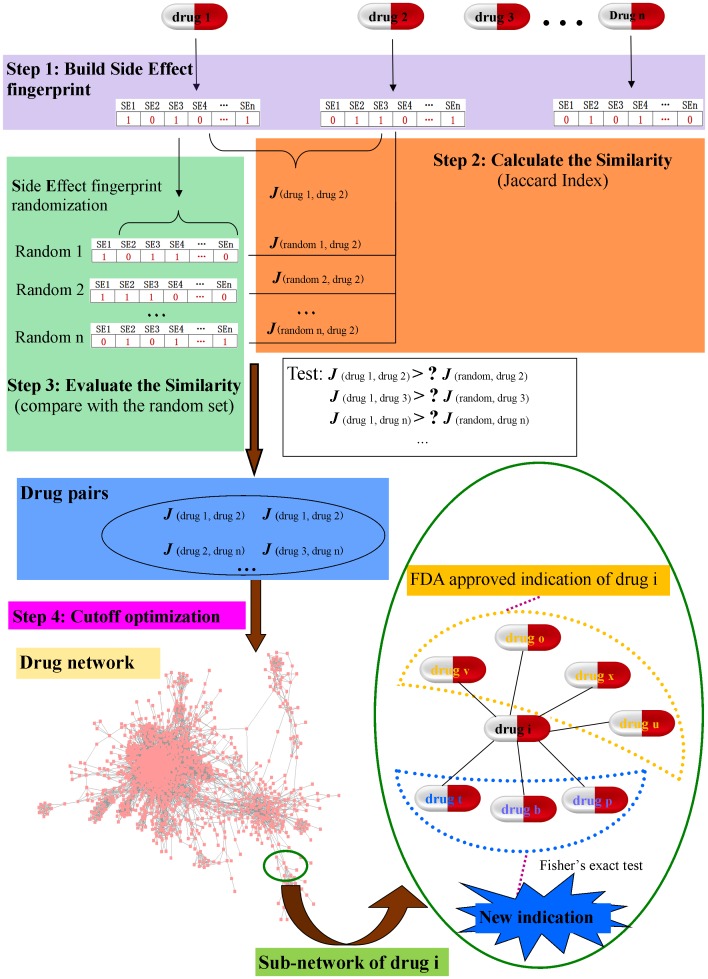
Construction of drug network using side effects.

The difference in the quantity of side effects associated with drugs may lead to similarity bias. It's necessary to evaluate whether the similarities between two drugs are randomly generated. As shown in **Figure 1**, the random side effect fingerprint sets of *drug A* were generated by randomly selecting the same number of side effects from the side effects pool 10000 times. For each random side effect fingerprint of *drug A*, the *J*(*random A, drug B*) similarity was calculated according to formula I. Then, the random Jaccard index set *S*(*drug A, drug B*) = {*J*(*random A 1,drug B*), *J*(*random A 2,drug B*),… *J*(*random A 10000, drug B*) between *drug A* and *drug B* was obtained. The *Z score* calculated by formula II was used to test whether the similarities between drug A and drug B were significantly larger than the random distribution. (*Z score*≥2.576 was set as the threshold)
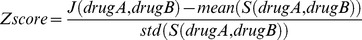
(II)


#### Step 4: Cutoff optimization

Cutoff optimization is an important process which could directly influence the specificity of the drug network. In this network, drugs sharing similar side effects were expected to be clustered together. Two criteria were considered when optimizing the threshold. First, the selected drug-drug pairs should cover as many drugs as possible. Second, the percentage of drug-drug pairs that share the same indication should be as high as possible. Usually, the two instances are contradictory to each other. When the cutoff is set at Jaccard index = 0, all of the drugs would be covered. A non-sense drug-drug network would be built with lots of totally unrelated drug-drug pairs. On the other hand, if the cutoff is set at Jaccard index = 1, then only a few drug-drug pairs would be selected.

### MeSH hierarchy and drug indication prediction

MeSH contains plentiful hierarchically arranged disease information, which makes it convenient to investigate drug indications. In this study, the potential indications of a drug could be predicted according to the MeSH hierarchy distribution of its neighbours (the 4^th^ level of MeSH hierarchy item could well represent an indication).

*EASE score* [26] is offered as a conservative adjustment to the Fisher exact probability, which was defined in DAVID [27] to evaluate gene-enrichment in pathways. The smaller the *EASE Score*, the higher the enrichment of genes in the pathways (see details in http://david.abcc.ncifcrf.gov/content.jsp?file=functional_annotation.html#fisher).

Herein, we used the *EASE score* to evaluate drug indication enrichment in the network. Taking *drug A* and *indication i* as an example, we built a 2×2 contingency table (See **Table 3**). Sorting the *EASE score in descending order*, we could generate the rank positions of indications for *drug A*.

**Table 3:** The 2×2 contingency table of *drug A* and indication *i*.

	*Drug A*'s neighbours	The background network
Indication *i*	n -1	r
Other indications	N-n	d-r

n: The number of *drug A*'s neighbors which are approved for indication *i* (n≥2);

N: The number of *drug A*'s neighbors;

r: the number of drugs in the network which are approved for indication *i*;

d: The number of drugs in the network.

Herein, the network represents the connected component which contains *drug A*. The connected component is defined as a sub-network in which any two drugs are connected to each other by paths.

### Performance evaluation

Normalized discounted cumulative gain (NDCG)[28] was originally used to evaluate web search engine algorithms in the field of information retrieval. It can measure the usefulness of a document based on its position in the result list.

Here we used NDCG to measure the effectiveness of ranking performance for each drug's predicted results (see formula III).
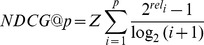
(III)


Z is the normalization constant.

*i* is the rank position of *indication m*.

*rel_i_* is the relevance value of *indication m*. If *indication m* is the FDA approved indication of *drug A*, *rel_i_* is set to 1, otherwise, *rel_i_* is set to 0.

p is the maximum position.

For example if the FDA approved indications of *drug A* are ranked at *2, 3* *&* *8*, respectively, while the ideal rank position of *drug A*'s FDA-approved indications should be *1, 2* *&* *3*,then_:_

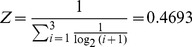



In the top 5 results,
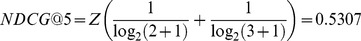



## Results

### Cutoff selection criteria and drug-drug network construction

**Figure 2:**
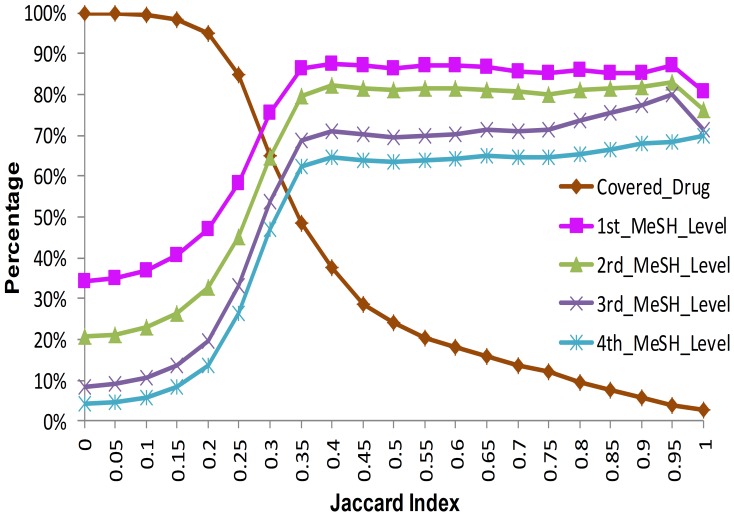
The trends of covered drugs and drug-drug pairs that share the same terms in the MeSH hierarchy.

As mentioned in **Methods**, we investigated variation trends in the percentage of covered drugs and co-indicated drug-drug pairs according to different Jaccard indexes. As shown in **Figure 2**, the percentage of co-indicated drug-drug pairs is positively correlated with the Jaccard index at four MeSH hierarchy levels. This evidence also suits our hypothesis that drugs with similar side effects would display similar functions. From **Figure 2**, it is obvious that the percentage of co-indicated pairs dramatically increases from 26.27% to 62.35% in the inflection area at the Jaccard index [0.2, 0.35] at the fourth level of MeSH hierarchy. Herein, Jaccard index = 0.275 was defined as the cutoff threshold. Then a drug-drug network based on side effect similarities was constructed, which contains 17400 drug-drug pairs, covering 1647 drugs. 1234 drugs with no less than 2 neighbors were used as our test samples. These drugs can be mapped to 81 ATC therapeutic categories including 5337 FDA approved drug-indication pairs, covering 584 indications. On average, 36.87% of the drugs achieved NDCG@5≥0.7, which means the known FDA-approved indications were well predicted. For details, the top 10 categories with NDCG@5≥0.7 were listed in **Table 4** (see full list in **Table S2**). The drugs for diabetes, obesity, laxatives and antimycobacterials were best predicted with more than 80% of them achieving NDCG@5≥0.7. The drugs for cardiovascular disease (diuretics and agents acting on the renin-angiotensin system) were also well predicted with more than 70% of them achieving NDCG@5≥0.7

**Table 4:** Top 10 ATC therapeutic categories with NDCG@5≥0.7.

ATC code	Therapeutic category	NO. drugs	Percentage of drugs with NDCG@5≥0.7
A10	Drugs used in diabetes	33	84.85%
A08	Antiobesity preparations, excluding diet products	12	83.33%
A06	Laxatives	6	83.33%
J04	Antimycobacterials	6	83.33%
G02	Other gynecologicals	11	72.73%
C09	Agents acting on the renin-angiotensin system	43	72.09%
J05	Antivirals for systemic use	37	70.27%
C03	Diuretics	38	68.42%
B05	Blood substitutes and perfusion solutions	3	66.67%
R07	Other respiratory system products	3	66.67%

Besides that, we also investigated the prediction performance in a different indication range (number of drugs approved for the indication) (see **Table 5**). Totally, 32.77% FDA approved drug-indication pairs could be predicted in the top 5 results, covering 202 (34.59%) indications. It seems that the indications with more drug approvals are likely to be predicted in the top 5 results. Good performance was generated in the indications with 30∼40 and 46∼55 approved drugs. The top 5 drug indication pairs in these groups achieved 40.69% and 61.33% of the corresponding FDA approved drug indication pairs respectively. In addition, for indications with 15 or more FDA drug approvals, more than 62.75% of them were ranked with at least one drug-indication pair at the top 5 positions.

**Table 5:** Prediction performance of the 1234 drugs in different indication range (number of network drugs approved for an indication).

NO. drugs per indication	NO. indications approved by FDA	Top 5 covered indications (percentage)	NO. drug-indication pairs approved by FDA	Top 5 covered drug-indication pairs (percentage)
less than 5	196	11 (5.61%)	430	18 (4.19%)
6∼10	130	26 (20.00%)	556	66 (11.87%)
11∼15	66	27 (40.91%)	508	92 (18.11%)
16∼20	66	32 (62.75%)	560	87 (15.54%)
21∼25	38	26 (68.42%)	566	124 (21.91%)
26∼30	20	16 (80.00%)	389	99 (25.45%)
31∼35	19	17 (89.47%)	412	185 (44.90%)
36∼40	14	13 (92.86%)	376	153 (40.69%)
41∼45	8	7 (87.50%)	224	58 (25.89%)
46∼50	5	5 (100.00%)	163	106 (65.03%)
51∼55	8	8 (100.00%)	297	183 (61.62%)
56∼60	4	4 (100.00%)	150	92 (61.33%)
more than 60	10	10 (100.00%)	706	486 (68.84%)
Total	584	202 (34.59%)	5337	1749 (32.77%)

Herein, only the FDA approved drug-indication pairs are considered a positive set. The prediction performance may be underestimated due to the fact that drug-indications have not yet been approved though they may well be capable of treating disease. For example, the drug-indications being tested in the clinical studies through phase I, II and III, were also classified as false positive. We manually checked the 1858 drug-indication pairs with an *EASE score*≤10^−5^ (see details in **Table S3**). We found that 80.73% of them could be verified by preclinical/clinical studies or scientific literature (see **Table 6**). We further investigated three drugs to understand their predicted results.

**Table 6:** Predicted results with evidence supported at an EASE score≤10^−5^.

Drug-indication pairs	Number	Percentage
FDA-approved	1336	71.91%
Clinical	132	7.10%
Preclinical	32	1.72%
Unknown	358	19.27%

### Dynastat

**Figure 3:**
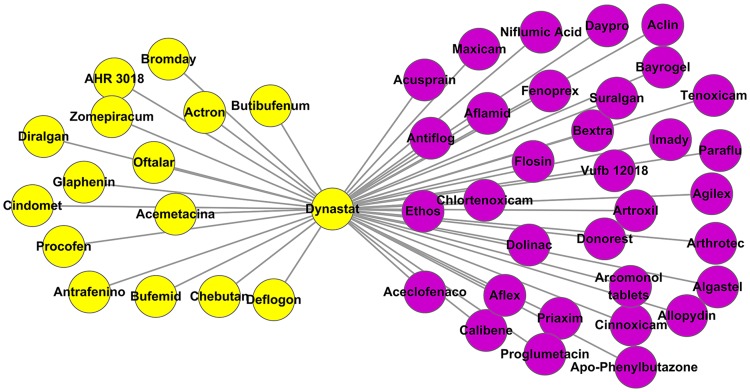
Sub-network of Dynastat. Each node represents a drug. Drugs approved for pain management are marked in yellow. Drugs approved for rheumatoid arthritis therapy are marked in purple.

Dynastat (also called Parecoxib) is a COX-2 selective inhibitor developed by Pfizer. It was initially approved for pain management by the European Union in 2002. Dynastat relieves pain through modulating prostaglandins levels. It exerts the effect by inhibiting COX-2, which is responsible for converting arachidonic acid to prostaglandin G2 and prostaglandin H2. Prostaglandins also play an important role in the pathogenesis of rheumatoid arthritis (RA) [29]. Prostaglandins were found at elevated levels in rheumatoid synovial fluid, and the bone-resorption activity produced by rheumatoid synovial tissues was shown to be mediated in part by prostaglandin E2 [30], which is one of the downstream products of prostaglandin H2 in the arachidonic acid metabolism pathway. In our predicted results, the neighbours of Dynastat are significantly enriched in pain relief (EASE score = 1.76×10^−14^) and rheumatoid arthritis (EASE score = 1.03×10^−21^) management. As shown in **Figure 3**, Dynastat displays a similar side effect profile to 33 RA drugs in addition to the pain drugs. Up to now, a group of COX-2 inhibitors were verified as promising drugs in the treatment of RA. For example, Celecoxib, Rofecoxib and Valdecoxib have already been approved in RA treatment. Considering that Parecoxib is a water-soluble prodrug that can be rapidly hydrolyzed into Valdecoxib [31], it may also be effective in the treatment of RA.

### Tasmar

**Figure 4:**
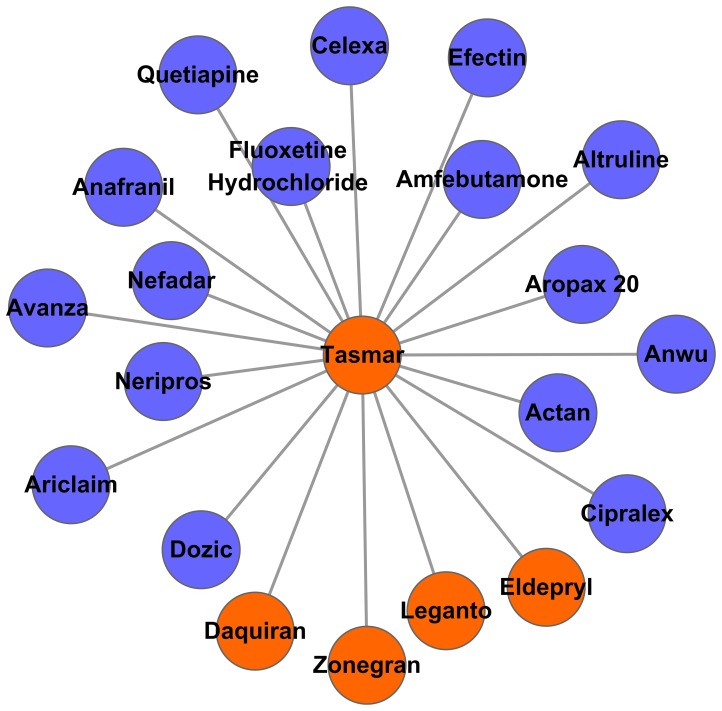
Sub-network of Tasmar. Each node represents a drug. Drugs approved for the treatment of Parkinson's disease are marked in orange. Drugs approved for rheumatoid arthritis therapy are marked in blue.

Tolcapone (brand name Tasmar) developed by Roche, was approved as an effective adjunctive treatment with Levodopa for Parkinson's disease in 1997 [32]. As a catechol-o-methyltransferase (COMT) inhibitor, Tasmar could improve the pharmacokinetic profile of Levodopa in two ways. First, it could directly inhibit the metabolic path from Levodopa to 3-O-methyldopa, which may finally increase the Levodopa half-life. Second, it may facilitate the transport of Levodopa to the brain by reducing 3-O-methyldopa, which may compete with Levodopa in brain barrier penetration [33]. The mechanism of actions also may explain why Tolcapone shares similar side effects with only 4 drugs for Parkinson therapy (*EASE score* = 0.39). We found that the indication of Tolcapone's neighbours was significantly enriched in anti-depression functions (15 drugs were approved for depression treatment, EASE score = 5.09×10^−8^. See **Figure 4**). A group of pharmaco-genetics studies showed that COMT variations are correlated with the effective management of depression [34], [35], [36], [37], [38], [39]. Besides that, Elin et al's study [40] proved that the Met-variants of COMT Val158Met are risk variants for depression in the Swedish population. All of the available evidence indicates that COMT inhibitors may have an effect on depression. In fact, the application of COMT inhibitors in depression treatment was approved for a US patent in 2005 (US 20050137162 A1). In animal studies, an increase of SAMe in the central nervous system was detected after the administration of Tolcapone [41]. SAMe is a naturally occurring compound with putative antidepressant properties [42]. This effect, coupled with antidepressant properties exhibited in the rat model of chronic mild stress-induced anhedonia [43], suggests that Tolcapone may have significant antidepressant properties. Moreover, in an open study on 21 adults with major depressive disorders, the group treated with Tolcapone showed significant improvement over the placebo group (17-item Hamilton Rating Scale for Depression 19.4+/−2.9 vs 10.7+/−5.5; Clinical Global Impressions Severity 3.9+/−0.6 vs 2.4+/−1.1; Beck Depression Inventory 21.6+/−8.1 vs 12.3+/−8.6) [44]. The preliminary results suggest that Tolcapone may be a promising anti-depressant.

### Adamon

**Figure 5:**
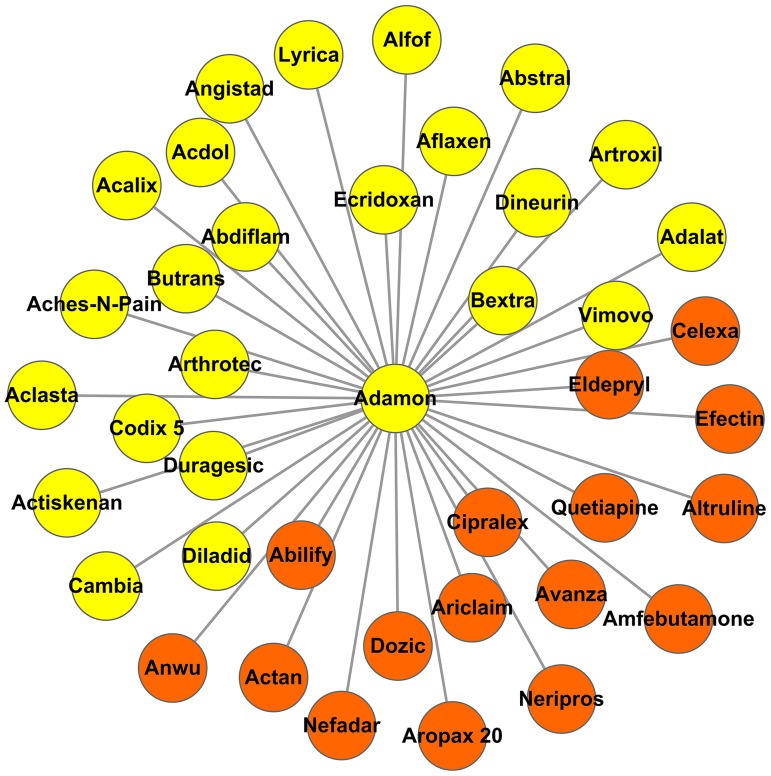
Sub-network of Adamon. Each node represents a drug. Drugs approved for the treatment of Parkinson 's disease are marked in orange. Drugs approved for pain treatment are marked in blue.

Tramadol hydrochloride (brand name Adamon) is a centrally acting synthetic analgesic used to treat moderately severe pain, which was first approved in 1977 as a product of the German pharmaceutical company Grünenthal GmbH. It is supposed to have an analgesic effect on pain based on two complementary mechanisms of action derived from its affinity for the mu opioid receptor and its blockade of norepinephrine and serotonin reuptake [45], [46]. Herein, two systems involved in pain relief are activated by Tramadol; namely, the opioid and the descending monoaminergic pain modulating pathways. In our predicted results, it shares similar side effect profiles with 22 pain management drugs (general pain: EASE score = 0.021; postoperative pain: *EASE score = 0.0063*). As shown in **Figure 5**, Tramadol is also connected to 13 anti-depression drugs (*EASE score* = 9.06×10^−5^). Desmeules's study [47] suggested that the analgesic action of Tramadol is mainly related to the central monoaminergic mechanism rather than opioid receptor pathways. Antidepressants usually act by inhibiting norepinephrine-serotonin reuptake, which is similar to Tramadol's effect of blocking monoaminergic reuptake [48]. In addition, opioid systems are also influenced in the pathophysiology of depression [49]. All the evidence suggests that Tramadol may have an effect on depression. In fact, a group of preclinical studies based on several depressive mice models showed the efficacy of Tramadol in depression management, such as the forced swimming test and the tail suspension test [48], [50], [51]. In 2008, the application of Tramadol in depression treatment was patented by European Union (EP20080011241). More promisingly, according to the pipeline of e-Therapeutics, the indication of Tramadol on depression has already been moved to Phase IIb clinical studies http://www.etherapeutics.co.uk/Information/pipeline.html.

### External data evaluation

After we tested the predicted results of all drugs in the network, external drugs with side effect data were also tested. 98 drugs exclusively covered in SIDER were used as inputs to our system to calculate similarities with the drugs in the network one by one.

As described in the **Methods**, we calculated similarities between 98 SIDER drugs with the background 2183 drugs. As shown in **Table 7**, 84.69% of SIDER drugs showed similarities to the network drugs with Jaccard index≤0.275. Herein, the Jaccard index = 0.225 in the inflection area [0.2, 0.35] was set as the threshold. Next, 61 drugs with more than 2 neighbours were inputted to evaluate the method. Finally, the indications of 41 drugs could be predicted since they have more than 2 neighbouring drugs approved for the same indication. Among the top 5 predicted results, 36.59% of the drugs reached a performance score NDCG@5≥0.7. In addition, by selecting the top 5 predicted results of 41 drugs with an EASE score≤0.05, 106 drug- indication pairs were generated (See details in **Table S4**). During the manual check, we found that 50.94% of them were FDA approved or could be verified by clinical trials or scientific literature (See **Table 8**).

**Table 7:** The similarity of 98 SIDER drugs in test sample set with the drugs in the network.

Similarity (Jaccard index)	Drugs with more than 2 neighbours (Percentage)
[0,0.15)	5 (5.10%)
[0.15,0.2)	21 (21.43.%)
[0.2,0.225)	11 (11.22%)
[0.225,0.25)	25 (25.51%)
[0.25,0.275)	21 (21.43%)
More than 0.275	15 (15.31%)

**Table 8:** Predicted drug-indication pairs of SIDER drugs.

Drug-indication pairs	Number	Percentage
FDA-approved	37	34.91%
Clinical	10	9.43%
Preclinical	7	6.60%
Unknown	52	49.06%

## Discussion

In this study, we integrated drug side effect data from several different resources. With these data, we built a drug network and screened new indications of drugs based on their network neighbours. Our method performed well at predicting their FDA approved indications in the top 5 positions. In addition to screening new indications of FDA approved drugs, our method could also be extended to candidate drugs with clinical side effect data. Since approved drugs sharing similar side effect profiles with query candidates could be identified, the indications of candidates could be inferred by its neighbours. Especially for drugs which failed in the late clinical stages, the comprehensive side effect data should have been generated in early clinical studies. These side effects can be used as inputs to our repositioning platform so that new indications for these drugs can be predicted.

Our drug repositioning platform has its own limitations. Due to the differences between side effect data resources, during an external data test using 98 drugs exclusively covered by SIDER, we found 58.16% of these SIDER drugs have low side effect similarities with the drugs in the network. Our method is not applicable to these SIDER drugs. Since very few side effect databases are available, many efforts are still needed to build or integrate these resources. For example, clinical case reports or other adverse event reporting systems may supply additional information on drug side effects. Up to now, only the direct neighbours in our network were considered to evaluate the indications in the study. We could quantify the influences of each neighbouring drug in the indication enrichment process according to their side effect similarities. Besides that, considering the fact that side effects vary in severity and frequency of occurrence, the current Jaccard index may not correctly mimic side effect similarities between the drugs. For example, the severity of a given side effect might be tagged as “serious” or “mild”, while its frequency of occurrence might be described as “common” or “rare”. Actually, these two side effect parameters are also related to the number of clinical patient samples and drug doses in treatment. Further meta-analysis should be carried out to modify the side effect data in order to take account of information regarding the severity and frequency of these side effects. This could certainly improve the accuracy of the predictions.

Drug repositioning is a complicated process. It is impossible that any one computational method alone would be accurate enough to provide promising results. A package of methods from different perspectives could be integrated to make predictions more precise. In our study, we also compared our results to Lun's [21] predicted results. In his study, 14040 drug-indication pairs are proposed. 14.57% of them overlapped with our results (747 drug-indications with EASE score≤0.05, see details in **Table S5**). In particular, five of the drugs (Ziprasidone, Quetiapine, Oxcarbazepine, Clozapine, Sildenafil) mentioned in Lun's paper were able to be used in the treatment of obsessive-compulsive disorder. The associations were also predicted by our method with a low EASE score≤6.35×10^−3^. The overlapping results from different methods, which may integrate comprehensive features in drug repositioning at the chemical levels, the MOA levels, and the phenotypic levels could provide promising predictions as well as cross validations.

## Supporting Information

Table S1 **The 98 drugs exclusively covered in SIDER.**
(XLSX)

Table S2 **Performance of drugs in 81 ATC therapeutic categories.**
(XLSX)

Table S3 **The predicted 1858 drug-indication pairs with an** ***EASE score*** **≤10^−5^.**
(XLSX)

Table S4 **The predicted 106 drug-indication pairs in external data evaluation using SIDER.**
(XLSX)

Table S5 **The 747 drug-indication pairs overlapped with Lun**'**s paper.**
(XLSX)
